# Estimated Fiscal Effects of Medicare Advantage’s Quartile Payment System

**DOI:** 10.1001/jamahealthforum.2023.4030

**Published:** 2023-12-08

**Authors:** Roslyn C. Murray, David J. Meyers, Erin C. Fuse Brown, Andrew M. Ryan

**Affiliations:** 1 Department of Health Management & Policy, University of Michigan, Ann Arbor; 2 Department of Health Services, Policy & Practice, Brown University, Providence, Rhode Island; 3 Georgia State University College of Law, Georgia State University, Atlanta, Georgia

## Abstract

This economic analysis estimates fiscal effects of the quartile adjustments made to Medicare Advantage payments as part of the Patient Protection and Affordable Care Act.

## Introduction

The Patient Protection and Affordable Care Act (ACA) introduced a quartile-based payment system to encourage more Medicare Advantage (MA) plan offerings, better benefits, and greater enrollment.^1^ This system classifies counties into quartiles based on historical traditional Medicare spending, providing payment boosts to areas with lower spending. The highest-spending counties (quartile 4) receive a 95% adjustment to their MA payments, increasing to 100% in quartile 3, 107.5% in quartile 2, and 115% in quartile 1.^2^ The current debate about payment in MA is focused on the unintended payment differences between MA and traditional Medicare (eg, risk adjustment), but it is also important to quantify intentional payment differences. Therefore, we used national data from January 2013 to December 2021 to estimate the fiscal impact of the quartile adjustments.

## Methods

This economic analysis used data from the MA ratebooks and the Geographic Variation Public Use File. First, we identified quartile adjustments for each county in each year and the proportion of MA beneficiaries subject to each adjustment. We calculated the proportion of additional payments due to the adjustments compared with a counterfactual in which all plans were paid 100% of historical traditional Medicare spending. We also identified the average monthly benchmark, which determines base payments, weighted by county enrollment. We excluded counties subject to pre-ACA benchmark caps and counties with benchmarks that were only partially subject to the quartile system (“phase-in counties”) from January 2012 to December 2016. Data were analyzed using Stata, version 17. We followed the CHEERS reporting guideline. The University of Michigan institutional review board deemed the project exempt because it used publicly accessible, aggregate data. Informed consent was not possible for the analysis.

## Results

Our sample included 20 447 county-years from 3215 counties. In all, 35.6% of MA beneficiaries were in counties with a 95% adjustment in 2013, declining to 22.7% in 2021; 15.5% were in counties with a 115% adjustment in 2013, increasing to 20.7% in 2021 (Figure). The estimated fiscal impact of the quartile adjustments increased over the study period due to a shift in MA enrollment toward higher adjustment quartiles and an overall increase in MA enrollment compared with traditional Medicare and benchmark levels. The quartile system generated an estimated $796.7 million in added payments in 2013, increasing to $11.9 billion in 2021 and totaling $46.7 billion in additional payments since its inception (Table).

**Figure.**
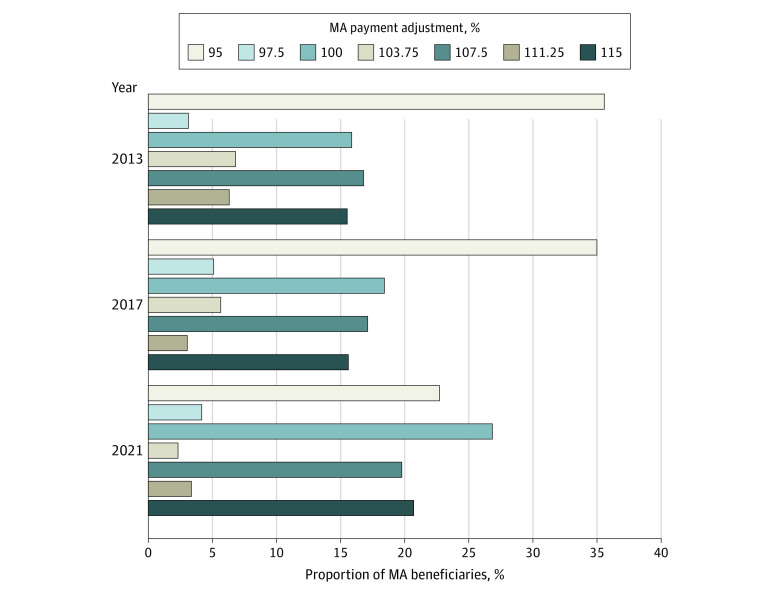
Estimated Proportion of Medicare Advantage (MA) Beneficiaries Subject to Varying Levels of Payment Adjustment in 2013, 2017, and 2021 Estimates exclude counties that had benchmarks capped at pre–Patient Protection and Affordable Care Act (ACA) levels and counties where only a portion of the benchmarks were determined by the quartile adjustments (2013-2016). All counties received a blended rate between the pre-ACA benchmark and the quartile adjustments in 2012. There were 9 possible adjustments, because some counties transitioned quartiles each year. The figure does not include the proportion of beneficiaries in counties that received a 101.25% or a 105% adjustment because it was close to 0 in all years.

**Table.** Estimated Additional Payments to MA From the Quartile Payment System, 2013-2021^**a**^

Payment year	Estimated additional payment, %	Total MA beneficiaries	Average monthly benchmark, $	Estimated additional payment, $
2013	2.70	3 073 769	799.74	796 715 840
2014	3.55	3 926 385	816.00	1 362 971 008
2015	2.48	12 667 710	765.04	2 886 793 728
2016	2.29	12 328 815	796.97	2 699 351 552
2017	2.32	18 972 858	823.38	4 339 774 976
2018	2.84	20 299 272	849.41	5 883 908 096
2019	3.22	21 619 094	899.33	7 522 951 168
2020	3.45	23 627 700	949.23	9 280 254 976
2021	3.82	26 518 194	980.56	11 906 810 880
Total	NA	143 033 797	NA	46 679 531 520

Abbreviations: ACA, Patient Protection and Affordable Care Act; MA, Medicare Advantage; NA, not applicable.

^a^ Estimates exclude counties that had benchmarks capped at pre-ACA levels and counties where only a portion of the benchmarks were determined by the quartile adjustments (2013-2016). All counties received a blended rate between the pre-ACA benchmark and the quartile adjustments in 2012. The total summed estimated additional payments are not precise due to precision issues in the statistical analysis software.

## Discussion

The quartile system led to an estimated $46.7 billion more in payments to MA plans from 2013 to 2021 than would have occurred if benchmarks were set at 100% of historical traditional Medicare spending. A key concern is what would be lost if MA payments were cut. Prior work found that small adjustments to MA benchmarks have little effect on the number and quality of plans offered.^3,4,5^

To our knowledge, this is the first estimate of fiscal effects of eliminating MA adjustments and instead setting benchmarks based on 100% of traditional Medicare spending. The Medicare Payment Advisory Commission’s proposal to replace the adjustments, increase rebates, integrate a discount factor, and eliminate the pre-ACA cap is estimated to save $2 billion annually.^1^

One limitation is that we assumed no behavioral effects of the quartile system: if the adjustments led to greater MA enrollment, our estimates could be inflated. However, small changes in MA payments were not associated with changes in enrollment, suggesting that quartile adjustments had little, if any, effect on enrollment.^4,5^ Instead, our estimates are likely conservative because we did not include phase-in counties, account for quality bonuses, or account for additional risk coding in MA that increases plan payments above 100% of traditional Medicare spending. Eliminating the quartile system through congressional action may generate savings that could be reinvested into Medicare and is unlikely to significantly harm MA enrollees.^3,4,5^

## Notes

Supplement. Data Sharing Statement
