# Supplementary material for: Estimated Fiscal Effects of Medicare Advantage’s Quartile Payment System
Source: JAMA Health Forum. 2023 Dec 8;4(12):e234030. doi: 10.1001/jamahealthforum.2023.4030 (PMC10709768; doi:10.1001/jamahealthforum.2023.4030)
Supplement: Supplement. — Data Sharing Statement [file jamahealthforum-e234030-s001.pdf]

Data Sharing Statement

Murray. Estimated Fiscal Effects of Medicare Advantage's Quartile Payment System. *JAMA Health Forum*. Published December 08, 2023. doi:10.1001/jamahealthforum.2023.4030

Data
Data available: No
